# Incense Use and Cardiovascular Mortality among Chinese in Singapore: The Singapore Chinese Health Study

**DOI:** 10.1289/ehp.1307662

**Published:** 2014-08-15

**Authors:** An Pan, Maggie L. Clark, Li-Wei Ang, Mimi C. Yu, Jian-Min Yuan, Woon-Puay Koh

**Affiliations:** 1 Saw Swee Hock School of Public Health, and; 2 Yong Loo Lin School of Medicine, National University of Singapore and National University Health System, Singapore; 3 Department of Environmental and Radiological Health Sciences, Colorado State University, Fort Collins, Colorado, USA; 4 Epidemiology and Disease Control Division, Ministry of Health, Singapore; 5 Keck School of Medicine, University of Southern California, Los Angeles, California, USA; 6 Division of Cancer Control and Population Sciences, University of Pittsburgh Cancer Institute, Pittsburgh, Pennsylvania, USA; 7 Department of Epidemiology, Graduate School of Public Health, University of Pittsburgh, Pittsburgh, Pennsylvania, USA; 8 Duke-NUS Graduate Medical School Singapore, Singapore

## Abstract

Background: Incense burning is common in many parts of the world. Although it is perceived that particulate matter from incense smoke is deleterious to health, there is no epidemiologic evidence linking domestic exposure to cardiovascular mortality.

Objective: We examined the association between exposure to incense burning and cardiovascular mortality in the Singapore Chinese Health Study.

Methods: We enrolled a total of 63,257 Singapore Chinese 45–74 years of age during 1993–1998. All participants were interviewed in person to collect information about lifestyle behaviors, including the practice of burning incense at home. We identified cardiovascular deaths via record linkage with the nationwide death registry through 31 December 2011.

Results: In this cohort, 76.9% were current incense users, and most of the current users (89.9%) had burned incense daily for ≥ 20 years. Relative to noncurrent users, current users had a 12% higher risk of cardiovascular mortality [multivariable adjusted hazard ratio (HR) = 1.12; 95% CI: 1.04, 1.20]. The HR was 1.19 (95% CI: 1.03, 1.37) for mortality due to stroke and 1.10 (95% CI: 1.00, 1.21) for mortality due to coronary heart disease. The association between current incense use and cardiovascular mortality appeared to be limited to participants without a history of cardiovascular disease at baseline (HR = 1.16; 95% CI: 1.07, 1.26) but not linked to those with a history (HR = 1.00; 95% CI: 0.86, 1.17). In addition, the association was stronger in never-smokers (HR = 1.12; 95% CI: 1.02, 1.23) and former smokers (HR = 1.19; 95% CI: 1.00, 1.42) than in current smokers (HR = 1.05; 95% CI: 0.91, 1.22).

Conclusions: Long-term exposure to incense burning in the home environment was associated with an increased risk of cardiovascular mortality in the study population.

Citation: Pan A, Clark ML, Ang LW, Yu MC, Yuan JM, Koh WP. 2014. Incense use and cardiovascular mortality among Chinese in Singapore: The Singapore Chinese Health Study. Environ Health Perspect 122:1279–1284; http://dx.doi.org/10.1289/ehp.1307662

## Introduction

It has been widely acknowledged that ambient air pollution is a significant risk factor for cardiovascular morbidity and mortality (Chen et al. 2008). However, with the exception of secondhand smoke exposure (Dunbar et al. 2013), the effect of indoor air pollution on the cardiovascular system is less clear. Growing evidence suggests that indoor biomass combustion from solid fuels is associated with elevated blood pressure (Baumgartner et al. 2011; Clark et al. 2011; Dutta et al. 2011; McCracken et al. 2007) and higher risk of coronary heart disease (CHD) (Lee et al. 2012). However, with social and economic development, the use of coal and other solid fuels has been decreasing in many countries. Very few studies have investigated incense burning as a source of indoor air pollution and a risk factor for cardiovascular disease (CVD). Incense burning at home for ritual or religious purpose is a common practice among Chinese populations in China (Tse et al. 2011), Taiwan (Liao et al. 2006), and Singapore (Friborg et al. 2008), as well as in populations of India (Dewangan et al. 2013) and Arabian Gulf countries (Cohen et al. 2013). Many studies on the composition of particulate matter (PM) from incense burning have identified airborne particles and associated organic components, which are potential air pollutants deleterious to health (Chiang and Liao 2006; Chiang et al. 2009; Chuang et al. 2013; Cohen et al. 2013; Dewangan et al. 2013; Fang et al. 2002, 2003; Ho and Yu 2002; Jetter et al. 2002; Lin et al. 2012; Lombardozzi et al. 2010; Lung and Hu 2003; Lung et al. 2003; Manoukian et al. 2013; Yang et al. 2012).

In a previous study using data from the Singapore Chinese Health Study, we found that incense use was significantly associated with an increased risk of upper respiratory tract cancer in a population-based cohort of middle-aged and elderly people in Singapore (Friborg et al. 2008). In the present study, we examined the association between incense use and cardiovascular mortality risk using the same cohort. We also differentiated between mortality due to CHD and stroke, given the potential differences in the pathophysiology of these two diseases (Hyvärinen et al. 2010; Wilhelmsen et al. 2005).

This cohort provides an opportunity to evaluate the association in a population in which incense use is very common for many households (Friborg et al. 2008), but the prevalence of outdoor air pollution (Velasco and Roth 2012) and indoor solid fuel use (Desai et al. 2004) is very low.

## Methods

*Study population*. We conducted this study using data from a population-based prospective cohort, the Singapore Chinese Health Study, which was established between April 1993 and December 1998. For this cohort, 63,257 Chinese adults, 45–74 years of age, were recruited from the residents in government-built housing estates, where 86% of the Singapore population resided during the period of recruitment (Hankin et al. 2001). The recruitment was limited to two major dialect groups of Chinese in Singapore, the Hokkien and the Cantonese, who originated from the contiguous provinces of Fujian and Guangdong, respectively, in the southern part of China. The institutional review board of the National University of Singapore approved this study, and all recruited participants gave informed consents.

*Data collection*. At recruitment, trained interviewers conducted face-to-face interviews using a structured, scanner-readable questionnaire and obtained information on demographics, height, weight, cigarette smoking, habitual physical activity, sleep hours, medical history (e.g., physician-diagnosed hypertension, diabetes, CHD, stroke), alcohol consumption, and dietary intake (by a validated 165-item food frequency questionnaire). For cigarette smoking, the participants were asked “Have you ever smoked at least one cigarette a day for 1 year or longer.” Participants were termed “never-smokers” if they answered “no”; “former smokers” if they answered “yes, but I quit smoking”; and “current smokers” if they answered “yes, and I currently smoke.” Former and current smokers were then asked about the number of cigarettes smoked per day, number of years of smoking, and number of years of quitting (only in former smokers).

No information was collected on passive smoking at baseline. However, during reinterview of 52,322 participants at the follow-up I visit conducted between 1999 and 2004 (Butler et al. 2006), participants provided responses on whether they were exposed to secondhand smoke at home or at work on a daily basis.

*Ascertainment of incense exposure*. At baseline, participants were asked whether their household ever burned incense (yes, no); if they answered “yes,” they were asked about the number of years they had burned incense (≤ 10 years, 11–20 years, 21–30 years, 31–40 years, or ≥ 41 years). Participants were asked if they had burned incense for the past 1 year; those who answered “yes” were defined as current users, and were further asked for the frequency of burning (a few times per year, a few times per month, a few times per week, or daily), as well as the placement of the altar (participant’s bedroom, other bedroom, living room, dining room, or kitchen) and the intensity of burning (during the night only, during the day only, intermittently during the day, or at all times). Information on incense use was not updated during subsequent follow-up interviews.

*Ascertainment of mortality*. We identified deaths through record linkage with the Singapore Registry of Births and Deaths. For the current analysis, we updated mortality data every year through 31 December 2011. By law in Singapore, all deaths in the country must be registered and reported to the Registry of Births and Deaths. Linkage is done by perfect matching of the unique National Identification Card Number and verified by name. The participants were recontacted in two follow-up visits: 1999–2004 (follow-up I) and 2006–2010. To our knowledge, only 47 participants from this cohort were lost to follow-up due to migration out of Singapore or for other reasons, suggesting that emigration among participants was negligible in this cohort and that vital statistics were virtually complete.

The main contributing causes of death were coded according to the *International Classification of Diseases, Ninth Revision*; codes 390–459 were used for cardiovascular deaths, codes 410–414 for CHD deaths, and codes 430–438 for deaths due to stroke. Codes for cardiovascular deaths include all CHD and other heart disease, stroke, hypertension, and diseases of arteries, arterioles, and capillaries.

*Statistical analysis*. We counted person-years from date of recruitment to either the date of death, loss to follow-up, or 31 December 2011, whichever occurred first. We used Cox proportional hazards regression methods to calculate the hazard ratio (HR) of incense use and cardiovascular mortality risk. All Cox regression models included the following covariates collected at recruitment: age, year of recruitment (1993–1995, 1996–1998), gender, dialect (Hokkien, Cantonese), education (no formal education, primary school, secondary school or above), body mass index (BMI; < 20.0, 20.0–23.9, 24.0–27.9, ≥ 28.0 kg/m^2^), alcohol consumption (none, monthly, weekly, daily), years of smoking (never, < 20, 20–39, ≥ 40 years), amount of smoking (never smoked, ≤ 12, 13–22, 23–32, ≥ 33 cigarettes/day), years since quitting smoking (never smoked, < 1 year, 1–4 years, 5–19 years, ≥ 20 years), moderate physical activity (< 0.5, 0.5–3.9, ≥ 4.0 hr/week), duration of sleep (hours per day), daily energy intake (kilocalories per day, quartiles), dietary intake of vegetables, fruits, polyunsaturated fatty acids (grams per day, quartiles); and self-reported history of physician-diagnosed cancer, hypertension, diabetes, CHD, and stroke. We performed tests for trend by entering ordinal categorical variables as continuous variables in the Cox regression models. Based on *a priori* hypotheses, we stratified the analysis by self-reported history of physician-diagnosed CHD/stroke as well as by smoking status (never, former, or current smoker) at baseline. We also tested interactions between incense use and gender, and education level using the likelihood ratio test, and we conducted stratified analysis if significant interactions were found. We also conducted a sensitivity analysis that included further adjustment for secondhand smoking among individuals who participated in the follow-up I visit (*n* = 52,322).

We estimated population attributable risk (PAR) based on the following formula:

*PAR*% = 100 × *P*_e_(*HR* – 1) ÷ [*P*_e_(*HR* – 1) + 1],

where *P*_e_ was the prevalence of the exposure (incense use) in the study population and the HR was derived from Cox regression models. Statistical analysis was conducted using SAS statistical software, version 9.1 (SAS Institute Inc., Cary, NC). All *p*-values were two-sided, and those < 0.05 were considered statistically significant.

## Results

Incense use was relatively common at baseline in this cohort of middle-aged and elderly Chinese living in Singapore, with 76.9% reporting current use of incense at home (77.4% in men and 76.4% in women), and another 13.1% reporting previous use but not in the past 1 year (former users, *n* = 8,259). Only 10% of the cohort members were never-users. Among 48,620 current users, most had used it daily for > 20 years (89.9%), the altar was primarily in the living room (91.9%), and most participants kept incense burning intermittently during the day (80.8%) (see Supplemental Material, Table S1). As shown in Table 1, the current incense users were more likely to speak Hokkien dialect (55.8%) than Cantonese (44.2%). Compared with noncurrent users, current users were less educated, more likely to be ever-smokers, and less likely to be physically active. They also had a lower dietary intake of fruits and polyunsaturated fatty acids. Otherwise, both groups were similar in age, BMI, gender distribution, prevalence of self-reported history of physician-diagnosed comorbidities (hypertension, CHD, stroke, and cancer), intake of alcohol, and daily duration of sleep.

**Table 1:** Baseline characteristics of cohort members by current use of incense in the Singapore Chinese Health Study.

Characteristic	Current users (*n *= 48,620)	Noncurrent users (*n *= 14,637)
Age at baseline (years)	56.5 ± 8.0	56.4 ± 8.2
BMI (kg/m^2^)	23.2 ± 3.3	22.9 ± 3.2
Gender
Men	21,647 (44.5)	6,307 (43.1)
Women	26,937 (55.5)	8,330 (56.9)
Dialect
antonese	21,487 (44.2)	7,797 (53.3)
Hokkien	27,133 (55.8)	6,840 (46.7)
Education
No formal education	15,000 (30.8)	2,333 (15.9)
Primary school (1–6 years)	22,985 (47.3)	5,065 (34.6)
≥ Secondary school	10,635 (21.9)	7,329 (49.5)
Cigarette smoking
Never	32,976 (67.8)	10,954 (74.8)
Former	5,346 (11.0)	1,647 (11.3)
urrent	10,298 (21.2)	2,036 (13.9)
Alcohol consumption
None/monthly	42,925 (88.3)	13,021 (88.9)
Weekly	3,940 (8.1)	1,166 (8.0)
Daily	1,755 (3.6)	450 (3.1)
Moderate physical activity (hr/week)
< 0.5	38,444 (79.1)	10,828 (74.0)
0.5–3.9	6,406 (13.2)	2,382 (16.3)
≥ 4.0	3,770 (7.7)	1,427 (9.7)
History of disease
Diabetes mellitus	4,486 (9.2)	1,210 (8.3)
Hypertension	11,481 (23.6)	3,573 (24.4)
oronary heart disease	1,942 (4.0)	656 (4.5)
Stroke	706 (1.5)	241 (1.7)
ancer	1,440 (3.0)	496 (3.4)
Sleep duration (hr/day)	7.0 ± 1.1	7.0 ± 1.1
Dietary intake (g/day)
Vegetables and related juices	109.0 ± 62.8	115.6 ± 65.8
Fruits and related juices	192.9 ± 163.8	234.1 ± 182.3
Fiber	12.4 ± 5.7	13.7 ± 6.0
Polyunsaturated fat	8.6 ± 4.7	9.7 ± 5.2
Data are presented as mean ± SD or *n* (%). There were no missing data for these variables.		

During a mean (± SD) follow-up time of 14.7 ± 4.1 years, we documented 5,043 cases of cardiovascular deaths in this cohort, including 2,851 CHD deaths and 1,381 fatal stroke cases. The mean (± SD) age at death was 72.4 ± 8.3 years for CVD, 72.1 ± 8.2 years for CHD, and 73.0 ± 8.3 years for stroke.

Table 2 shows the HRs of cardiovascular mortality by incense use. The adjusted HR was increased in current users but not in former users, compared with never-users. For stroke mortality, both never-users and former users had similar risk. Thus, in subsequent analyses, we grouped never-users and former users together as noncurrent users. After adjusting for established and potential risk factors of CVD, current incense users had a significant 12% increase in risk of cardiovascular mortality [HR = 1.12; 95% confidence interval (CI): 1.04, 1.20] compared with noncurrent users. In particular, we estimated a 10% higher risk of CHD mortality (HR = 1.10; 95% CI: 1.00, 1.21) and a 19% increase in stroke mortality (HR = 1.19; 95% CI: 1.03, 1.37) in current users. Taking into consideration both the frequency and duration of incense use, there was a statistically significant 12% increase in risk estimated for daily exposure of ≥ 20 years compared with noncurrent users (HR = 1.12; 95% CI: 1.04, 1.21), but associations were not significant in other groups. Estimated associations were similar for daily exposure of 20–40 years and > 40 years, and the vast majority (87%) of those who reported using incense for > 20 years had actually done so for > 40 years. Hence, we combined these two groups in subsequent analyses.

**Table 2:** Incense use and cardiovascular mortality in the Singapore Chinese Health Study (1993–2011).^*a*^

Exposure	Participants (*n*)	Person-years	CVD		CHD		Stroke	
			Deaths	HR (95% CI)	Deaths	HR (95% CI)	Deaths	HR (95% CI)
Status of incense use
Never use	6,378	95,964	485	1.00	277	1.00	122	1.00
Former use	8,259	119,400	502	0.91 (0.80, 1.03)	286	0.90 (0.76, 1.06)	131	0.98 (0.77, 1.26)
Current use	48,620	711,397	4,056	1.06 (0.96, 1.16)	2,288	1.03 (0.91, 1.18)	1,128	1.18 (0.97, 1.43)
*p* for trend				0.03		0.21		0.03
Noncurrent use	14,637	215,364	987	1.00	563	1.00	253	1.00
Current use	48,620	711,937	4,056	1.12 (1.04, 1.20)	2,288	1.10 (1.00, 1.21)	1,128	1.19 (1.03, 1.37)
Frequency and duration of incense use
Noncurrent use	14,637	215,364	987	1.00	563	1.00	253	1.00
Current, less than daily use	3,574	52,580	289	1.04 (0.91, 1.18)	156	0.98 (0.82, 1.17)	80	1.12 (0.87, 1.45)
Current, daily use for ≤ 20 years	1,313	20,156	64	1.08 (0.83, 1.39)	33	0.99 (0.70, 1.41)	18	1.11 (0.69, 1.80)
Current, daily use for > 20 years	43,733	638,660	3,703	1.12 (1.04, 1.21)	2,099	1.11 (1.01, 1.23)	1,030	1.20 (1.04, 1.38)
*p* for trend				0.002		0.01		0.01
Current, daily use for 20–40 years	5,867	91,508	403	1.11 (0.99, 1.25)	226	1.08 (0.92, 1.27)	114	1.22 (0.97, 1.54)
Current, daily use for > 40 years	37,866	547,152	3,300	1.12 (1.04, 1.21)	1,873	1.12 (1.01, 1.23)	916	1.20 (1.04, 1.38)
^***a***^The estimates were generated using Cox proportional hazards models, with adjustment for age at recruitment; year of recruitment (1993–1995, 1996–1998); gender; dialect (Hokkien, Cantonese); education (no formal education, primary school, ≥ secondary school); BMI (< 20.0, 20.0–23.9, 24.0–27.9, ≥ 28.0 kg/m^2^); alcohol consumption (none, monthly, weekly, daily); years of smoking (never, < 20 years, 20–39 years, ≥ 40 years); amount of smoking (never, ≤ 12, 13–22, 23–32, ≥ 33 cigarettes/day); years since quitting smoking (never, < 1 year, 1–4 years, 5–19 years, ≥ 20 years); moderate physical activity (< 0.5, 0.5–3.9, ≥ 4.0 hr/week); duration of sleep (hr/day); daily energy intake (kcal/day); dietary intake of vegetables, fruits, fiber, poly­unsaturated fatty acids (grams/day, quartiles); and self-reported history of physician-diagnosed hypertension, diabetes, cancer, CHD, and stroke.								

We estimated a significant interaction between incense use and baseline CHD/stroke history for cardiovascular mortality (*p* for interaction = 0.02) but not for CHD or stroke mortality (*p* = 0.15 and *p* = 0.28, respectively) (Table 3). Current incense use was positively associated with cardiovascular mortality in participants without a baseline history of CHD and stroke (HR = 1.16; 95% CI: 1.07, 1.26) but not in those with a baseline history of CHD or stroke (HR = 1.00; 95% CI: 0.86, 1.17). No significant interaction was found between incense use and gender (*p* for interaction = 0.24 for cardiovascular mortality).

**Table 3:** Incense use and cardiovascular mortality in the Singapore Chinese Health Study, stratified by baseline history of CVD, smoking, or education level at baseline (1993–2011).^*a*^

Exposure	Participants	Person-years	CVD		CHD		Stroke	
			Deaths	HR (95% CI)	Deaths	HR (95% CI)	Deaths	HR (95% CI)
Stratified by baseline CHD or stroke^*b*^
Without baseline CHD or stroke
Noncurrent use	13,774	204,917	757	1.00	421	1.00	203	1.00
urrent use	46,082	681,316	3,378	1.16 (1.07, 1.26)	1,847	1.13 (1.01, 1.26)	972	1.24 (1.06, 1.45)
With baseline CHD or stroke
Noncurrent use	863	10,447	230	1.00	142	1.00	50	1.00
urrent use	2,538	30,081	678	1.00 (0.86, 1.17)	441	1.04 (0.85, 1.27)	156	1.06 (0.76, 1.48)
Stratified by smoking status^*c*^
Never-smokers
Noncurrent use	10,954	165,511	582	1.00	314	1.00	160	1.00
urrent use	32,976	499,294	2,144	1.12 (1.02, 1.23)	1,137	1.10 (0.96, 1.25)	672	1.26 (1.05, 1.51)
Former smokers
Noncurrent use	1,647	22,125	169	1.00	100	1.00	37	1.00
urrent use	5,346	71,726	651	1.19 (1.00, 1.42)	407	1.25 (1.00, 1.57)	154	1.18 (0.81, 1.71)
Current smokers
Noncurrent use	2,036	27,728	236	1.00	149	1.00	56	1.00
urrent use	10,298	140,377	1,261	1.05 (0.91, 1.22)	744	1.00 (0.83, 1.20)	302	1.03 (0.77, 1.38)
Stratified by education level^*d*^
No formal education
Noncurrent use	2,333	33,063	272	1.00	151	1.00	74	1.00
urrent use	15,000	215,540	1,650	0.98 (0.86, 1.11)	884	0.93 (0.78, 1.11)	519	1.13 (0.88, 1.45)
Primary school
Noncurrent use	5,065	73,625	383	1.00	223	1.00	94	1.00
urrent use	22,985	336,371	1,844	1.14 (1.02, 1.27)	1,068	1.10 (0.95, 1.27)	484	1.30 (1.04, 1.63)
≥ Secondary school
Noncurrent use	7,239	108,676	332	1.00	189	1.00	85	1.00
urrent use	10,635	159,485	562	1.25 (1.09, 1.44)	336	1.28 (1.06, 1.53)	125	1.17 (0.88, 1.55)
^***a***^The *p* for interactions between incense use (binary variable) and baseline CHD/stroke (binary variable) were 0.02, 0.15, and 0.28 for CVD, CHD, and stroke mortality, respectively. The *p* for interactions between incense use (binary variable) and smoking status (never, former, and current smokers) were 0.27, 0.21, and 0.21 for CVD, CHD, and stroke mortality, respectively. The *p* for interactions between incense use (binary variable) and education level (no formal education, primary school, and ≥ secondary school) were 0.03, 0.03, and 0.81 for CVD, CHD, and stroke mortality, respectively. ^***b***^The estimates were generated using Cox proportional hazards models, with adjustment for age at recruitment; year of recruitment; gender; dialect; education; BMI; alcohol consumption; years of smoking; amount of smoking; years since quitting smoking; moderate physical activity; duration of sleep; daily energy intake; dietary intake of vegetables, fruits, fiber, and polyunsaturated fatty acids; and self-reported history of physician-diagnosed hypertension, diabetes, and cancer. ^***c***^The estimates were generated using Cox proportional hazards models, with adjustment for age at recruitment; year of recruitment; gender; dialect; education; BMI; alcohol consumption; moderate activity; duration of sleep; daily energy intake; dietary intake of vegetables, fruits, fiber, and polyunsaturated fatty acids; and self-reported history of physician-diagnosed hypertension, diabetes, cancer, CHD, and stroke. ^***d***^The estimates were generated using Cox proportional hazards models, with adjustment for age at recruitment; year of recruitment; gender; dialect; BMI; alcohol consumption; years of smoking; amount of smoking; years since quitting smoking; moderate physical activity; duration of sleep; daily energy intake; dietary intake of vegetables, fruits, fiber, and poly­unsaturated fatty acids; and self-reported history of physician-diagnosed hypertension, diabetes, cancer, CHD, and stroke.								

The HRs of cardiovascular mortality adjusted for all covariates except cigarette smoking and education level were biased away from the null (HR = 1.20; 95% CI: 1.12, 1.29 for current use compared with nonuse), indicating that smoking and education level were major confounding factors in our analysis (see Supplemental Material, Table S2). In analysis stratified by smoking status (Table 3), current incense use was associated with a 12% (HR = 1.12; 95% CI: 1.02, 1.23) and a 19% (HR = 1.19; 95% CI: 1.00, 1.42) increase in risk of cardiovascular mortality in never-smokers and formers smokers, respectively. Conversely, the association was weak and not statistically significant in current smokers (HR = 1.05; 95% CI: 0.91, 1.22; *p* for interaction = 0.27). In analysis stratified by education level (Table 3), the associations persisted in those with primary school (HR = 1.14; 95% CI: 1.02, 1.27) and ≥ secondary school education (HR = 1.25; 95% CI: 1.09, 1.44) but not in those without formal education (HR = 0.98; 95% CI: 0.86, 1.11; *p* for interaction = 0.03).

When we repeated our analysis with never-users of incense as the reference group, associations with the frequency and duration of incense use were attenuated for cardiovascular and CHD mortality [HRs for current daily use > 20 years: 1.06 (95% CI: 0.97, 1.17) and 1.05 (95% CI: 0.92, 1.10), respectively] but were similar for stroke mortality (HR for current daily use > 20 years: 1.19; 95% CI: 0.98, 1.44) (see Supplemental Material, Table S3). The HRs for former use and current use (compared with never use) that were stratified by history of CVD at baseline, smoking status, and education level were generally consistent with HRs for current versus noncurrent use (see Supplemental Material, Table S4).

In a sensitivity analysis with further adjustment for secondhand smoking among the subgroup of individuals who participated in the follow-up I interview conducted between 1999 and 2004 (*n* = 52,322), the results were not materially changed (see Supplemental Material, Table S5). Compared with never-users of incense, there was a statistically significant 19% increase in risk of cardiovascular mortality for current incense users after adjusting for daily exposure to secondhand smoking (HR = 1.19; 95% CI: 1.04, 1.36).

Given the high prevalence of incense use in this population, we further calculated the PAR of incense use for the outcomes. In this cohort, we estimated that 7.1% of CHD deaths and 12.1% of stroke deaths could be attributed to current daily use of incense over a period of > 20 years. Among never-smokers, the estimated PARs for CHD and stroke deaths increased to 8.6% and 15.8%, respectively.

## Discussion

In this large population-based cohort of middle-aged and elderly Chinese adults in Singapore, we found that long-term exposure to incense burning at home on a daily basis for > 20 years was associated with increased risk of cardiovascular mortality. Incense use was associated with both CHD and stroke mortality based on models adjusted for multiple lifestyle and comorbidity factors, and associations were primarily present among never- and former smokers, those without a baseline history of CVD, and those with ≥ primary school education.

Worldwide, global industrialization has resulted in an increase in air pollution levels. Hence, for the last few decades, ambient air pollution has been studied for its deleterious effects on health, including the impact on CVD, the leading cause of death in industrialized countries (Chen et al. 2008). In a review of all studies published between 1950 and 2007, Chen et al. (2008) found suggestive evidence that ambient air pollution exposure may be a risk factor for cardiovascular mortality and that exposure to PM_2.5_ (PM ≤ 2.5 μm in aerodynamic diameter) was associated with an increased risk of cardiovascular mortality (range, 12–14% per 10-μg/m^3^ increase). With regard to stroke mortality, several time-series studies have estimated small but substantially significant associations with short-term elevated air pollution (including measures of PM, nitrogen dioxide, carbon monoxide, and ozone) (Brook et al. 2010). For long-term exposure, estimates from the Women’s Health Initiative showed significant increases in risk of both nonfatal (HR = 1.28; 95% CI: 1.02, 1.61) and fatal stroke (HR = 1.83; 95% CI: 1.11, 3.00) per 10-μg/m^3^ increase in PM_2.5_ (Miller et al. 2007). Cohort studies in Europe reported that outdoor air pollution from road traffic was positively associated with fatal stroke (Andersen et al. 2012) and mortality in stroke survivors (Maheswaran et al. 2010). Therefore, our findings are generally consistent with findings from studies of ambient air pollution.

Although many previous studies have focused on outdoor air pollution, few have explored the relation between cardiovascular outcomes and indoor air quality (Mortimer et al. 2012). One study in rural India (Dutta et al. 2011) and one study in rural China (Baumgartner et al. 2011) reported that biomass fuel use was positively associated with blood pressure. A randomized intervention study in Guatemalan women reported that transitioning from an open fire to an improved biomass stove was associated with reductions in blood pressure and ST segment depression (McCracken et al. 2007, 2011). A recent cross-sectional analysis of > 14,000 Chinese adults suggested that household air pollution from solid fuel use was positively associated with hypertension, diabetes, CHD, and stroke, after adjustment for potential confounders (Lee et al. 2012). An early cohort study of 957 men in Shanghai, China, reported that compared with gas fuel, indoor air pollution from coal fumes was associated with an increased risk of stroke mortality during a 12-year follow-up period (HR = 2.55; 95% CI: 1.30, 5.03) (Zhang et al. 1988). As described above, these previous studies focused mainly on solid fuel use but not on the other sources of indoor air pollutants.

To our knowledge, our study is the first to provide epidemiologic evidence that chronic exposure to daily incense burning in the home environment may increase the risk of cardiovascular mortality. Incense burning for ritual or religious purposes is a common practice in many countries and regions. For example, in a study in the United Arab Emirates, Yeatts et al. (2012) found that 86% of individuals used incense at least once a week and 44% of them used it daily. A study in Hong Kong reported a prevalence of 55–65% for incense use (Tse et al. 2011), and in the present study we found a prevalence of 77% for current incense use. Many studies have analyzed emissions from the burning of incense and identified volatile organic compounds and PM that could be deleterious to health (Chiang and Liao 2006; Chiang et al. 2009; Chuang et al. 2013; Cohen et al. 2013; Dewangan et al. 2013; Fang et al. 2002, 2003; Ho and Yu 2002; Jetter et al. 2002; Lin et al. 2012; Lombardozzi et al. 2010; Lung and Hu 2003; Lung et al. 2003; Manoukian et al. 2013; Yang et al. 2012). This includes fine PM that could raise the indoor concentrations of PM_2.5_ to concentrations far exceeding the outdoor standards specified by the World Health Organization (Dewangan et al. 2013; Fang et al. 2002; Jetter et al. 2002; Lee and Wang 2004), as well as volatile compounds that include polycyclic aromatic hydrocarbons, oxygenated monoterpenes, esters, and formaldehydes (Chiang and Liao 2006; Cohen et al. 2013; Ho and Yu 2002; Lombardozzi et al. 2010; Lung and Hu 2003; Lung et al. 2003; Manoukian et al. 2013; Yang et al. 2012). In a study in Taiwan, Liao et al. (2006) estimated similar size-integrated source emission rates between cooking and incense burning, and each contributed about 30% of indoor particle levels for particle sizes of 0.5–5 μm.

In the present study, we found that the association between incense use and cardiovascular mortality was weaker in current smokers than in never-smokers and former smokers. We hypothesize that this occurred because current smokers are already exposed to the pollutants from cigarette smoke, and incense burning confers little additional harmful effects on the vascular system on top of cigarette smoking. Previous studies comparing the quantity of PM and the emission of aerosol particles and gaseous pollutants have reported that indoor air pollution from incense burning is comparable to that from cigarette smoking (Dewangan et al. 2013; Miller et al. 2007). In fact, Mannix et al. (1996) found that burning incense could generate even larger quantities of PM (an average of > 45 mg/g burned) than burning cigarettes (~ 10 mg/g burned).

In an experimental study conducted on rats, Alokail et al. (2011) found that long-term exposure to incense smoke was associated with adverse metabolic changes, that is, increased triglyceride concentrations, decreased HDL (high-density lipoprotein) cholesterol concentrations, and a transient increase of leptin levels. An *in vitro* study by Lin et al. (2008) showed that the size and composition of particles from incense burning in temples were both important factors in inducing cytokine production and reducing nitric oxide formation in human coronary artery endothelial cell cultures. Cohen et al. (2013) reported that the expression levels of inflammatory biomarkers were increased in human lung epithelial cells exposed to smoke components formed from incense burning. Incense particles also had substantial oxidative potential as measured using the plasmid scission assay as an *in vitro* marker for the oxidative potential (Chuang et al. 2013). In addition, other mechanisms that may link exposure to ambient PM with CVD include the initiation of low-grade inflammation (Pope et al. 2004), induction of vascular thrombogenesis via the effects of PM on blood coagulability and vasospasm (Louwies et al. 2013; Lucking et al. 2008; Peters et al. 1997), dysregulation of cardiac autonomic system that leads to arrhythmias (Liao et al. 2009; Pope et al. 1999, 2004), and higher resting cerebrovascular resistance and lower cerebral blood flow velocity (Wellenius et al. 2013).

The strengths of the present study include its population-based design, large number of relatively homogenous participants, detailed information on lifestyle factors that were established or potential risk factors of cardiovascular mortality, and medical history obtained through in-person interviews. All exposure information, including self-reported history of physician-diagnosed comorbidities, was collected on an average of 8.9 years prior to outcomes. We had virtually complete ascertainment of data on mortality given that, to date, the number of participants lost to follow up was negligible. There are several unique characteristics of this population that made the study exceptional: *a*) The use of incense was quite common in the cohort, and the majority of the participants had used incense for > 20 years; *b*) Singapore is a small country with very low levels of outdoor air pollution (Velasco and Roth 2012); and *c*) there was almost no solid fuel use as recorded in a World Health Organization report (Desai et al. 2004). Taken together, the other sources of air pollution were therefore negligible or similar for all participants in this cohort. Thus, we had the unique opportunity to evaluate the association between incense use and cardiovascular mortality in this population.

Our findings should be interpreted with some limitations kept in mind. First, we measured incense exposure only at recruitment, and no information was captured during the follow-up. However, given that the majority of exposed individuals reported long-term exposure on a daily basis, and that the burning of incense is related to religious or ritual practices, the number of participants who had changed their habits subsequently would be small. Second, the medical conditions were self-reported at baseline, and we did not have accurate information on incident CVD during the follow-up period. Third, we did not measure the exact indoor air pollution status for each household, and we did not collect the information on the type of incense used or other practices that may influence indoor air pollution (e.g., opening of windows, use of air conditioners). Nevertheless, because of the prospective design of the study, the measurement errors and misclassifications of exposure are mostly nondifferential in nature in relation to the outcome of interest. Hence, we believe that any impact due to change in incense exposure or imperfect measure would likely result in an underestimation of the risk estimate. Finally, due to the observational nature of the study, residual or unmeasured confounding is still possible, and causality should be inferred with caution. Because incense use is related to religious and cultural practice, it is possible that study participants who use incense differ in other characteristics from those who do not burn incense, resulting in potential unmeasured confounders in our analysis.

## Conclusion

To our knowledge, the present study is the first to provide epidemiologic evidence that long-term exposure to indoor air pollution from incense burning may substantially contribute to the risk of cardiovascular mortality at the population level. We estimate that approximately 8% of CHD deaths and 12% of stroke deaths in the study population could be attributed to incense use. Considering the worldwide prevalence of incense burning, findings of this study have significant public health implications. Burning incense at home is linked to religious and ritual practice, and it is not our intention to discourage this activity. However, it is important to educate users on approaches to limit adverse health effects. Future studies should be undertaken to identify the least harmful types of incense as well as strategies to reduce exposure and improve indoor air quality when using incense.

## Supplemental Material

(266 KB) PDF
