# Supplementary material for: Incense Use and Cardiovascular Mortality among Chinese in Singapore: The Singapore Chinese Health Study
Source: Environ Health Perspect. 2014 Aug 15;122(12):1279–84. doi: 10.1289/ehp.1307662 (PMC4256695; doi:10.1289/ehp.1307662)
Supplement: (266 KB) PDF [file ehp.1307662.s001.508.pdf]

Supplemental Material

Incense Use and Cardiovascular Mortality among Chinese in Singapore: The Singapore Chinese Health Study

An Pan, Maggie L. Clark, Li-Wei Ang, Mimi C. Yu, Jian-Min Yuan, and Woon-Puay Koh

Table S1. Incense use practices among the current incense users in the Singapore Chinese Health Study (1993-2011).

	**Current users**	**Former users**
n	48,620	8,259
Duration of incense use		
≤ 10 years	404 (0.8)	249 (3.0)
11-20 years	1073 (2.2)	1399 (16.9)
21-30 years	2508 (5.2)	3286 (39.8)
31-40 years	3828 (7.9)	1191 (14.4)
≥ 40 years	40807 (83.9)	2134 (25.8)
Main altar placed in the home		
Master bedroom	88 (0.2)	NA
Other bedroom	117 (0.2)	NA
Living room	44677 (91.9)	NA
Dining room	590 (1.2)	NA
Kitchen	3148 (6.5)	NA
Intensity of burning		
During the day only	5340 (11.0)	NA
During the night only	2270 (4.7)	NA
Intermittently during the day	39291 (80.8)	NA
At all times	1719 (3.5)	NA

Data are shown as n (%). NA: not applicable.

Table S2. Alternative models of incense use and cardiovascular mortality in the Singapore Chinese Health Study (1993-2011).

Exposure	Persons	Deaths	Person-years	Crude model	Model 1[a]	Model 2[b]	Model 3[c]
Never users	6378	485	95964	1.00 (reference)	1.00 (reference)	1.00 (reference)	1.00 (reference)
Former users	8259	502	119400	0.85 (0.75, 0.96)	0.93 (0.82, 1.05)	0.92 (0.81, 1.05)	0.91 (0.80, 1.03)
Current users	48620	4056	711397	1.14 (1.04, 1.25)	1.15 (1.05, 1.27)	1.09 (0.99, 1.20)	1.06 (0.96, 1.16)
p for trend				< 0.001	< 0.001	0.006	0.03
Non-current users				1.00 (reference)	1.00 (reference)	1.00 (reference)	1.00 (reference)
Current users	48620	4056	711397	1.24 (1.16, 1.33)	1.20 (1.12, 1.29)	1.14 (1.06, 1.22)	1.12 (1.04, 1.20)

[a]Model 1: the estimates were generated using Cox proportional hazards models, with adjustment for age at recruitment, year of recruitment (1993-1995, 1996-1998), gender, dialect (Hokkien, Cantonese), body mass index (< 20.0, 20.0-23.9, 24.0-27.9, ≥ 28.0 kg/m^2), alcohol drinking (none, monthly, weekly, daily), moderate activity (< 0.5, 0.5-3.9, ≥ 4.0 hours/week), duration of sleep (hours/day), daily energy intake (kcal/day), dietary intakes of vegetables, fruits, fiber, polyunsaturated fatty acids (grams/day, quartiles), as well as self-reported history of physician-diagnosed hypertension, diabetes, cancer, coronary heart disease and stroke. [b]Model 2: the estimates were generated using Cox proportional hazards models, with adjustment for variables in model 1 plus education (no formal education, primary school, secondary school or higher). [c]Model 3: the estimates were generated using Cox proportional hazards models, with adjustment for variables in model 2 plus years of smoking (never, < 20 years, 20-39 years, ≥ 40 years), dose of smoking (never, ≤ 12, 13-22, 23-32, ≥ 33 cigarettes/day), years since quitting smoking (never, < 1 year, 1-4 years, 5-19 years, ≥ 20 years).

Table S3. Duration and frequency of incense use and cardiovascular mortality in the Singapore Chinese Health Study (1993-2011): never users as the reference group.[a]

Exposure	Persons	Person-years	CVD deaths	CVD HR (95% CI)	CHD deaths	CHD HR (95% CI)	Stroke deaths	Stroke HR (95% CI)
Never use	6378	95964	485	1.00	277	1.00	122	1.00
Former use	8259	119400	502	0.91 (0.80, 1.03)	286	0.90 (0.76, 1.06)	131	0.98 (0.77, 1.26)
Current, less than daily use	3574	52580	289	0.98 (0.85, 1.14)	156	0.92 (0.75, 1.12)	80	1.11 (0.83, 1.47)
Current, daily use for up to 20 years	1313	20156	64	1.03 (0.80, 1.34)	33	0.94 (0.66, 1.36)	18	1.13 (0.69, 1.85)
Current, daily use for > 20 years	43733	638660	3703	1.06 (0.97, 1.17)	2099	1.05 (0.92, 1.19)	1030	1.19 (0.98, 1.44)
p for trend				0.004		0.04		0.02

CHD: coronary heart disease; CVD: cardiovascular disease; CI: confidence interval; HR: hazard ratio.

[a]The estimates were generated using Cox proportional hazards models, with adjustment for age at recruitment, year of recruitment (1993-1995, 1996-1998), gender, dialect (Hokkien, Cantonese), education (no formal education, primary school, secondary school or higher), body mass index (BMI; < 20.0, 20.0-23.9, 24.0-27.9, ≥ 28.0 kg/m^2), alcohol drinking (none, monthly, weekly, daily), years of smoking (never, < 20 years, 20-39 years, ≥ 40 years), dose of smoking (never, ≤ 12, 13-22, 23-32, ≥ 33 cigarettes/day), years since quitting smoking (never, < 1 year, 1-4 years, 5-19 years, ≥ 20 years), moderate activity (< 0.5, 0.5-3.9, ≥ 4.0 hours/week), duration of sleep (hours/day), daily energy intake (kcal/day), dietary intakes of vegetables, fruits, fiber, polyunsaturated fatty acids (grams/day, quartiles), as well as self-reported history of physician-diagnosed hypertension, diabetes, cancer, CHD and stroke.

Table S4. Incense use and risk of cardiovascular mortality in the Singapore Chinese Health Study: stratified by history of coronary heart disease or stroke, smoking status, and education level at baseline with never users as the reference group (1993-2011).

Exposure	Persons	Person-years	CVD deaths	CVD HR (95% CI)	CHD deaths	CHD HR (95% CI)	Stroke deaths	Stroke HR (95% CI)
Stratified by baseline CHD or stroke[a]								
Without baseline CHD or stroke								
Never use	5977	91129	377	1.00	210	1.00	99	1.00
Former use	7797	113787	380	0.90 (0.78, 1.04)	211	0.89 (0.73, 1.08)	104	0.97 (0.74, 1.28)
Current use	46082	681316	3378	1.10 (0.98, 1.22)	1847	1.06 (0.92, 1.23)	972	1.22 (0.99, 1.51)
With baseline CHD or stroke								
Never use	401	4835	108	1.00	67	1.00	23	1.00
Former use	462	5613	122	0.97 (0.75, 1.26)	75	0.94 (0.68, 1.31)	27	1.03 (0.59, 1.80)
Current use	2538	30081	678	0.99 (0.80, 1.22)	441	1.01 (0.77, 1.32)	156	1.08 (0.68, 1.69)
Stratified by smoking status[b]								
Never smokers								
Never use	4845	74956	300	1.00	160	1.00	80	1.00
Former use	6109	90555	282	0.90 (0.76, 1.06)	154	0.91 (0.73, 1.14)	80	1.00 (0.73, 1.36)
Current use	32976	499294	2144	1.06 (0.94, 1.20)	1137	1.05 (0.88, 1.24)	672	1.26 (0.99, 1.59)
Former smokers								
Never use	613	8309	70	1.00	44	1.00	14	1.00
Former use	1034	13816	99	0.98 (0.72, 1.34)	56	0.87 (0.59, 1.30)	23	1.15 (0.59, 2.24)
Current use	5346	71726	651	1.17 (0.91, 1.50)	407	1.15 (0.83, 1.58)	154	1.26 (0.72, 2.20)
Current smokers								
Never use	920	12699	115	1.00	73	1.00	28	1.00
Former use	1116	15029	121	0.89 (0.69, 1.15)	76	0.90 (0.65, 1.24)	28	0.83 (0.49, 1.41)
Current use	10298	140377	1261	0.99 (0.81, 1.20)	744	0.94 (0.74, 1.20)	302	0.94 (0.63, 1.39)
Stratified by education level[c]								
No formal education								
Never use	1110	15835	137	1.00	79	1.00	32	1.00
Former use	1223	17228	135	0.89 (0.70, 1.13)	72	0.81 (0.58, 1.11)	42	1.24 (0.78, 1.97)
Current use	15000	215540	1650	0.92 (0.77, 1.10)	884	0.84 (0.66, 1.06)	519	1.27 (0.89, 1.82)

Exposure	Persons	Person-years	CVD deaths	CVD HR (95% CI)	CHD deaths	CHD HR (95% CI)	Stroke deaths	Stroke HR (95% CI)
Primary school level								
Never use	2107	31338	175	1.00	101	1.00	46	1.00
Former use	2958	42286	208	0.91 (0.74, 1.11)	122	0.91 (0.70, 1.19)	48	0.84 (0.56, 1.25)
Current use	22985	336371	1844	1.08 (0.92, 1.26)	1068	1.04 (0.85, 1.28)	484	1.18 (0.87, 1.60)
Secondary school level or above								
Never use	3161	48791	173	1.00	97	1.00	44	1.00
Former use	4078	59886	159	0.93 (0.75, 1.16)	92	0.94 (0.70, 1.25)	41	1.04 (0.67, 1.59)
Current use	10635	159485	562	1.21 (1.01, 1.44)	336	1.24 (0.98, 1.56)	125	1.19 (0.83, 1.69)

CHD: coronary heart disease; CVD: cardiovascular disease; CI: confidence interval; HR: hazard ratio.

[a]The estimates were generated using Cox proportional hazards models, with adjustment for age at recruitment, year of recruitment, gender, dialect, education, body mass index, alcohol drinking, years of smoking, dose of smoking, years since quitting smoking, moderate activity, duration of sleep, daily energy intake, dietary intakes of vegetables, fruits, fiber, polyunsaturated fatty acids, self-reported history of physician-diagnosed hypertension, diabetes, and cancer. [b]The estimates were generated using Cox proportional hazards models, with adjustment for age at recruitment, year of recruitment, gender, dialect, education, body mass index, alcohol drinking, moderate activity, duration of sleep, daily energy intake, dietary intakes of vegetables, fruits, fiber, polyunsaturated fatty acids, self-reported history of physician-diagnosed hypertension, diabetes, cancer, CHD and stroke. [c]The estimates were generated using Cox proportional hazards models, with adjustment for age at recruitment, year of recruitment, gender, dialect, body mass index, alcohol drinking, years of smoking, dose of smoking, years since quitting smoking, moderate activity, duration of sleep, daily energy intake, dietary intakes of vegetables, fruits, fiber, polyunsaturated fatty acids, self-reported history of physician-diagnosed hypertension, diabetes, cancer, CHD and stroke.

Table S5. Incense use and cardiovascular mortality in the Singapore Chinese Health Study: further adjustment for secondhand smoking (1999-2011).[a]

Exposure	Persons	Person-years	CVD deaths	CVD HR (95% CI)	CHD deaths	CHD HR (95% CI)	Stroke deaths	Stroke HR (95% CI)
Total participants								
Never incense users	5243	84263	245	1.00	141	1.00	60	1.00
Former incense users	7016	106414	283	0.99 (0.83, 1.17)	159	0.96 (0.76, 1.20)	70	1.03 (0.73, 1.46)
Current incense users	40063	625520	2241	1.19 (1.04, 1.36)	1284	1.19 (1.00, 1.42)	583	1.29 (0.98, 1.69)
Participants with exposure to secondhand smoking								
Never incense users	903	14589	35	1.00	21	1.00	9	1.00
Former incense users	1206	18396	35	0.83 (0.52, 1.32)	22	0.86 (0.47, 1.56)	9	1.88 (0.35, 2.23)
Current incense users	11086	173574	569	1.25 (0.89, 1.77)	319	1.17 (0.75, 1.83)	160	1.42 (0.72, 2.81)
Participants without exposure to secondhand smoking								
Never incense users	4340	69673	210	1.00	120	1.00	51	1.00
Former incense users	5810	88018	248	1.01 (0.84, 1.21)	137	0.97 (0.76, 1.24)	61	1.06 (0.73, 1.54)
Current incense users	28977	451947	1672	1.17 (1.01, 1.36)	965	1.19 (0.98, 1.45)	423	1.25 (0.93, 1.68)

CHD: coronary heart disease; CI: confidence interval; HR: hazard ratio.

[a]The analyses were conducted among 52,322 participants who participated in follow-up I interview (1999-2004) and provided information on secondhand smoking at home or at work on a daily basis. The estimates were generated using Cox proportional hazards models, with adjustment for age at recruitment, year of recruitment (1993-1995, 1996-1998), gender, dialect (Hokkien, Cantonese), education (no formal education, primary school, secondary school or higher), body mass index (BMI; < 20.0, 20.0-23.9, 24.0-27.9, ≥ 28.0 kg/m^2), alcohol drinking (none, monthly, weekly, daily), years of smoking (never, < 20 years, 20-39 years, ≥ 40 years), dose of smoking (never, ≤ 12, 13-22, 23-32, ≥ 33 cigarettes/day), years since quitting smoking (never, < 1 year, 1-4 years, 5-19 years, ≥ 20 years), moderate activity (< 0.5, 0.5-3.9, ≥ 4.0 hours/week), duration of sleep (hours/day), daily energy intake (kcal/day), dietary intakes of vegetables, fruits, fiber, polyunsaturated fatty acids (grams/day, quartiles), self-reported history of physician-diagnosed hypertension, diabetes, CHD and stroke, as well as exposure to secondhand smoking (yes or no; except in the stratified analysis).
